# Methyl (*Z*)-2-[(4-bromo-2-formyl­phen­oxy)meth­yl]-3-(4-methyl­phen­yl)acrylate

**DOI:** 10.1107/S1600536812006630

**Published:** 2012-02-17

**Authors:** S. Vijayakumar, S. Murugavel, D. Kannan, M. Bakthadoss

**Affiliations:** a Department of Physics, Sri Balaji Chokkalingam Engineering College, Arni, Thiruvannamalai 632 317, India; b Department of Physics, Thanthai Periyar Government Institute of Technology, Vellore 632 002, India; c Department of Organic Chemistry, University of Madras, Maraimalai Campus, Chennai 600 025, India

## Abstract

In the title compound, C_19_H_17_BrO_4_, the dihedral angle between the two benzene rings is 82.9 (2)°. The mol­ecular structure is stabilized by an intra­molecular C—H⋯O hydrogen bond, which generates an *S*(7) ring motif. The crystal packing is stabilized by C—H⋯O hydrogen bonds, which generate two centrosymmetic ring systems with *R_2_^2^*(18) and *R_2_^2^*(14) graph-set motifs. The crystal packing is further stabilized by inter­molecular π–π inter­actions [centroid–centroid distance = 3.984 (2) Å].

## Related literature

For background to the applications of acrylates, see: de Fraine & Martin (1991 ▶); Zhang & Ji (1992 ▶). For related structures, see: Wang *et al.* (2011 ▶); Vijayakumar *et al.* (2011 ▶). For hydrogen-bond motifs, see: Bernstein *et al.* (1995 ▶).
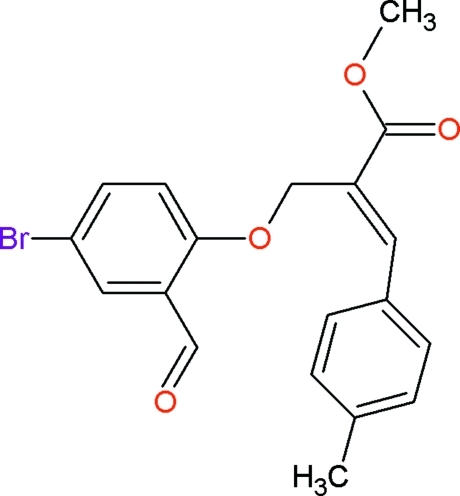


## Experimental

### 

#### Crystal data

C_19_H_17_BrO_4_
*M* *_r_* = 389.24
Triclinic, 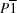

*a* = 7.9262 (4) Å
*b* = 8.9078 (5) Å
*c* = 13.2331 (6) Å
α = 74.387 (3)°
β = 83.593 (2)°
γ = 75.770 (3)°
*V* = 871.20 (8) Å^3^
*Z* = 2
Mo *K*α radiation
μ = 2.38 mm^−1^
*T* = 293 K
0.25 × 0.23 × 0.18 mm

#### Data collection

Bruker APEXII CCD diffractometer
Absorption correction: multi-scan (*SADABS*; Sheldrick, 1996 ▶) *T*_min_ = 0.546, *T*_max_ = 0.652
15446 measured reflections
3386 independent reflections
2419 reflections with *I* > 2σ(*I*)
*R*_int_ = 0.038

#### Refinement

*R*[*F* ^2^ > 2σ(*F* ^2^)] = 0.040
*wR*(*F* ^2^) = 0.121
*S* = 1.01
3386 reflections
219 parameters
H-atom parameters constrained
Δρ_max_ = 0.67 e Å^−3^
Δρ_min_ = −0.41 e Å^−3^

### 

Data collection: *APEX2* (Bruker, 2004 ▶); cell refinement: *APEX2* and *SAINT* (Bruker, 2004 ▶); data reduction: *SAINT* and *XPREP* (Bruker, 2004 ▶); program(s) used to solve structure: *SHELXS97* (Sheldrick, 2008 ▶); program(s) used to refine structure: *SHELXL97* (Sheldrick, 2008 ▶); molecular graphics: *ORTEP-3* (Farrugia, 1997 ▶); software used to prepare material for publication: *SHELXL97* and *PLATON* (Spek, 2009 ▶).

## Supplementary Material

Crystal structure: contains datablock(s) global, I. DOI: 10.1107/S1600536812006630/bt5816sup1.cif

Structure factors: contains datablock(s) I. DOI: 10.1107/S1600536812006630/bt5816Isup2.hkl

Supplementary material file. DOI: 10.1107/S1600536812006630/bt5816Isup3.cml

Additional supplementary materials:  crystallographic information; 3D view; checkCIF report

## Figures and Tables

**Table 1:** Hydrogen-bond geometry (Å, °)

*D*—H⋯*A*	*D*—H	H⋯*A*	*D*⋯*A*	*D*—H⋯*A*
C14—H14⋯O3	0.93	2.55	3.341 (3)	143
C4—H4⋯O2^i^	0.93	2.57	3.474 (4)	164
C18—H18⋯O1^ii^	0.93	2.49	3.388 (4)	161
C5—H5⋯O4^iii^	0.93	2.39	3.276 (4)	159

Symmetry codes: (i) 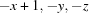; (ii) 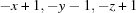; (iii) 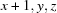.

**Fig. 1.**
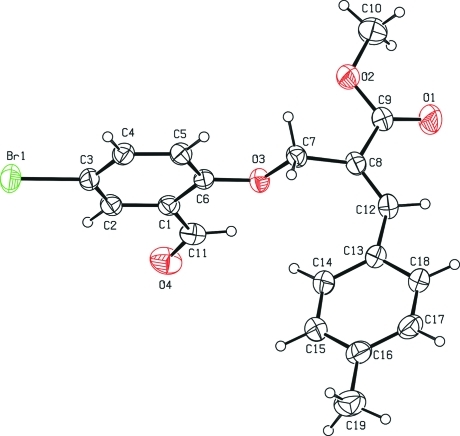
The molecular structure of the title compound with the atom numbering scheme. Displacement ellipsoids are drawn at the 30% probability level. H atoms are presented as a small spheres of arbitrary radius.

**Fig. 2.**
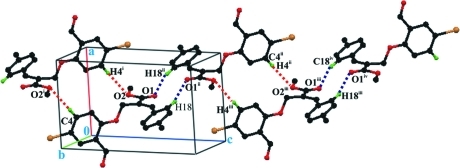
Part of the crystal structure of (I) showing C—H···O hydrogen bonds generating R22(18)(Red dotted lines) and R22(14) (blue dotted lines) centrosymmetric dimers, forming zigzag chains along [001]. [Symmetry codes:(i)1 - x, -y, -z; (ii)1 - x, -1 - y, 1 - z; (iii)x, -1 + y, 1 + z; (iv)1 - x, -2 - y, 2 - z].

**Fig. 3.**
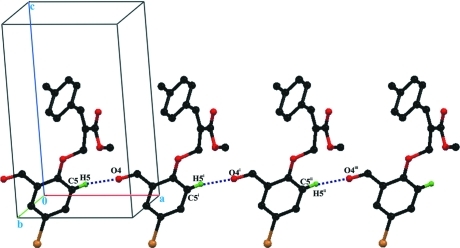
Part of the crystal structure of (I) showing C—H···O hydrogen bonds (blue dotted lines), with the formation of C(6) chains along [100]. [Symmetry codes: (i)1 + x, y, z; (ii)2 + x, y, z; (iii) 3 + x, y, z].

**Fig. 4.**
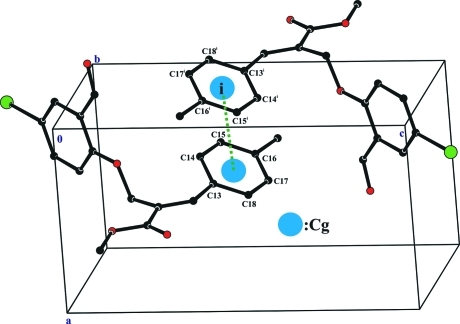
A view of the π—π interaction (dotted line) in the crystal structure of the title compound. Cg denotes centroid of the C13–C18 benzene ring. [Symmetry code: (i)-x, -y, 1 - z].

## supplementary crystallographic information

### Comment

Acrylate and its derivatives are important compounds because of their agrochemical and medical applications (de Fraine *et al.*, 1991; Zhang & Ji, 1992). We report herein the crystal structure of the title compound, (I).

Fig. 1. shows a displacement ellipsoid plot of (I), with the atom numbering scheme. The dihedral angle between the two benzene rings is 82.9 (2)°. The methyl acrylate (O1/O2/C7—C10) plane forms dihedral angles of 87.9 (1)° and 32.6 (1)° respectively, with the bromo formyl phenyl and methyl phenyl rings. The bromine atom deviates from the plane of the attached ring by -0.011 (1) Å. The geometric parameters of the title molecule agrees well with those reported for similar structures (Wang *et al.*, 2011, Vijayakumar *et al.*, 2011).

The molecular structure is stabilized by an intramolecular C14—H14···O3 hydrogen bond which generates an S(7) ring motif (Bernstein *et al.*, 1995) (Table 1). The crystal packing is stabilized by intermolecular C—H···O hydrogen bonds. The formation of the framework can be explained in terms of two-one substructures. In the first substructure C4—H4···O2 at (*x, y, z* and *1 - x, -y, -z*) and C18—H18···O1 at (*x, y, z* and *1 - x, -1 - y, 1 - z*) hydrogen bonding interactions form a cyclic centrosymmetric pattern, with the graph set motif *R_2_^2^*(18) and *R_2_^2^*(14), respectively. These combine to form zigzag chains which propagate along [001] (Fig. 2). In the second substructure, atom C5 in the molecule at (*x, y, z*) acts as a hydrogen bond donor to atom O4 in the molecule at (*1 + x, y, z*) generating C(6) chains which are running along [100] (Fig. 3). The crystal packing is further stabilized by an intermolecular π—π interactions with *Cg*—*Cg*^i^ separation of 3.984 (2) Å (Fig. 4; *Cg* is the centroid of the (C13–C18) benzene ring).

### Experimental

A solution of 5-bromo-2-hydroxybenzaldehyde (1.0 mmol, 0.201 g) and potassium carbonate (1.5 mmol, 0.207 g) in acetonitrile solvent (10 ml) was stirred for 15 minutes at room temperature. To this solution, (*Z*-methyl 2-(bromomethyl)-3-*p*-tolylacrylate (1.2 mmol, 0.324 g) was added dropwise till the addition is complete. After the completion of the reaction as indicated by TLC, acetonitrile was evaporated. Ethylacetate (15 ml) and water (15 ml) were added to the crude mass and extracted. The organic layer was dried over anhydrous sodium sulfate. Removal of solvent led to the crude product, which was purified through pad of silica gel (100–200 mesh) using ethylacetate and hexanes (1:9) as solvents. The pure title compound was obtained as a colorless solid (0.350 g, 90% yield). Single crystals suitable for X-ray diffraction were obtained by slow evaporation of a ethylacetate solution at room temperature.

### Refinement

All the H atoms were positioned geometrically, with C–H = 0.93–0.97 Å and constrained to ride on their parent atom, with *U*_iso_(H) =1.5*U*_eq_ for methyl H atoms and 1.2*U*_eq_(C) for other H atoms.

### Crystal data

**Table:**

C_19_H_17_BrO_4_	*Z* = 2
*M**_r_* = 389.24	*F*(000) = 396
Triclinic, *P*1	*D*_x_ = 1.484 Mg m^−^^3^
Hall symbol: -P 1	Mo *K*α radiation, λ = 0.71073 Å
*a* = 7.9262 (4) Å	Cell parameters from 3452 reflections
*b* = 8.9078 (5) Å	θ = 1.6–26.1°
*c* = 13.2331 (6) Å	µ = 2.38 mm^−^^1^
α = 74.387 (3)°	*T* = 293 K
β = 83.593 (2)°	Block, colourless
γ = 75.770 (3)°	0.25 × 0.23 × 0.18 mm
*V* = 871.20 (8) Å^3^	

### Data collection

**Table:**

Bruker APEXII CCD diffractometer	3386 independent reflections
Radiation source: fine-focus sealed tube	2419 reflections with *I* > 2σ(*I*)
Graphite monochromator	*R*_int_ = 0.038
Detector resolution: 10.0 pixels mm^-1^	θ_max_ = 26.1°, θ_min_ = 2.4°
ω scans	*h* = −9→9
Absorption correction: multi-scan (*SADABS*; Sheldrick, 1996)	*k* = −10→10
*T*_min_ = 0.546, *T*_max_ = 0.652	*l* = −16→16
15446 measured reflections	

### Refinement

**Table:**

Refinement on *F*^2^	Primary atom site location: structure-invariant direct methods
Least-squares matrix: full	Secondary atom site location: difference Fourier map
*R*[*F*^2^ > 2σ(*F*^2^)] = 0.040	Hydrogen site location: inferred from neighbouring sites
*wR*(*F*^2^) = 0.121	H-atom parameters constrained
*S* = 1.01	*w* = 1/[σ^2^(*F*_o_^2^) + (0.0704*P*)^2^ + 0.2283*P*] where *P* = (*F*_o_^2^ + 2*F*_c_^2^)/3
3386 reflections	(Δ/σ)_max_ = 0.001
219 parameters	Δρ_max_ = 0.67 e Å^−^^3^
0 restraints	Δρ_min_ = −0.41 e Å^−^^3^

### Special details

**Table:**

Geometry. All e.s.d.'s (except the e.s.d. in the dihedral angle between two l.s. planes) are estimated using the full covariance matrix. The cell e.s.d.'s are taken into account individually in the estimation of e.s.d.'s in distances, angles and torsion angles; correlations between e.s.d.'s in cell parameters are only used when they are defined by crystal symmetry. An approximate (isotropic) treatment of cell e.s.d.'s is used for estimating e.s.d.'s involving l.s. planes.
Refinement. Refinement of *F*^2^ against ALL reflections. The weighted *R*-factor *wR* and goodness of fit *S* are based on *F*^2^, conventional *R*-factors *R* are based on *F*, with *F* set to zero for negative *F*^2^. The threshold expression of *F*^2^ > σ(*F*^2^) is used only for calculating *R*-factors(gt) *etc*. and is not relevant to the choice of reflections for refinement. *R*-factors based on *F*^2^ are statistically about twice as large as those based on *F*, and *R*- factors based on ALL data will be even larger.

### Fractional atomic coordinates and isotropic or equivalent isotropic displacement parameters (Å^2^)

**Table:**

	*x*	*y*	*z*	*U*_iso_*/*U*_eq_	
C1	−0.0190 (3)	0.1409 (3)	0.0632 (2)	0.0436 (6)	
C2	−0.0529 (4)	0.2719 (3)	−0.0228 (2)	0.0492 (7)	
H2	−0.1652	0.3118	−0.0466	0.059*	
C3	0.0793 (4)	0.3418 (3)	−0.0723 (2)	0.0530 (8)	
C4	0.2463 (4)	0.2851 (3)	−0.0381 (2)	0.0521 (7)	
H4	0.3347	0.3345	−0.0727	0.063*	
C5	0.2831 (4)	0.1550 (3)	0.0472 (2)	0.0476 (7)	
H5	0.3958	0.1169	0.0706	0.057*	
C6	0.1509 (3)	0.0820 (3)	0.0978 (2)	0.0400 (6)	
C7	0.3452 (3)	−0.1066 (3)	0.2212 (2)	0.0463 (7)	
H7A	0.3805	−0.0227	0.2418	0.056*	
H7B	0.4283	−0.1395	0.1673	0.056*	
C8	0.3425 (4)	−0.2459 (3)	0.3141 (2)	0.0491 (7)	
C9	0.3846 (4)	−0.4096 (4)	0.2990 (3)	0.0568 (8)	
C10	0.4617 (6)	−0.5711 (5)	0.1796 (3)	0.0814 (11)	
H10A	0.5751	−0.6240	0.2056	0.122*	
H10B	0.4643	−0.5618	0.1055	0.122*	
H10C	0.3785	−0.6322	0.2151	0.122*	
C11	−0.1608 (4)	0.0680 (4)	0.1180 (3)	0.0555 (8)	
H11	−0.1325	−0.0241	0.1721	0.067*	
C12	0.3161 (4)	−0.2336 (4)	0.4127 (2)	0.0556 (8)	
H12	0.3271	−0.3307	0.4630	0.067*	
C13	0.2727 (4)	−0.0931 (4)	0.4554 (2)	0.0512 (7)	
C14	0.1771 (4)	0.0561 (4)	0.4051 (2)	0.0577 (8)	
H14	0.1347	0.0708	0.3395	0.069*	
C15	0.1444 (4)	0.1815 (4)	0.4507 (3)	0.0638 (9)	
H15	0.0807	0.2805	0.4151	0.077*	
C16	0.2035 (4)	0.1652 (4)	0.5481 (3)	0.0613 (8)	
C17	0.2973 (4)	0.0179 (4)	0.5982 (3)	0.0651 (9)	
H17	0.3392	0.0042	0.6637	0.078*	
C18	0.3305 (4)	−0.1087 (4)	0.5544 (3)	0.0627 (8)	
H18	0.3929	−0.2076	0.5911	0.075*	
C19	0.1688 (6)	0.3035 (5)	0.5977 (3)	0.0875 (12)	
H19A	0.1914	0.2644	0.6711	0.131*	
H19B	0.0493	0.3601	0.5898	0.131*	
H19C	0.2435	0.3744	0.5641	0.131*	
Br1	0.03444 (6)	0.52045 (4)	−0.19017 (3)	0.0788 (2)	
O1	0.3943 (5)	−0.5289 (3)	0.3685 (2)	0.0984 (9)	
O2	0.4125 (3)	−0.4137 (3)	0.19867 (17)	0.0616 (6)	
O3	0.1745 (2)	−0.0481 (2)	0.18098 (15)	0.0472 (5)	
O4	−0.3117 (3)	0.1191 (3)	0.0979 (2)	0.0792 (7)	

### Atomic displacement parameters (Å^2^)

**Table:**

	*U*^11^	*U*^22^	*U*^33^	*U*^12^	*U*^13^	*U*^23^
C1	0.0456 (16)	0.0445 (15)	0.0437 (15)	−0.0063 (12)	−0.0056 (12)	−0.0183 (12)
C2	0.0560 (17)	0.0468 (16)	0.0461 (16)	−0.0008 (14)	−0.0123 (14)	−0.0195 (13)
C3	0.079 (2)	0.0398 (15)	0.0375 (15)	−0.0019 (15)	−0.0099 (15)	−0.0129 (12)
C4	0.0628 (19)	0.0461 (16)	0.0462 (16)	−0.0129 (14)	0.0038 (14)	−0.0114 (13)
C5	0.0452 (16)	0.0483 (16)	0.0493 (16)	−0.0105 (13)	−0.0031 (13)	−0.0121 (13)
C6	0.0464 (16)	0.0381 (14)	0.0377 (13)	−0.0084 (12)	−0.0045 (12)	−0.0131 (11)
C7	0.0401 (15)	0.0445 (16)	0.0497 (16)	−0.0047 (12)	−0.0049 (12)	−0.0076 (13)
C8	0.0465 (16)	0.0432 (16)	0.0522 (17)	−0.0058 (12)	−0.0060 (13)	−0.0053 (13)
C9	0.065 (2)	0.0443 (17)	0.0537 (18)	−0.0052 (14)	−0.0029 (15)	−0.0060 (14)
C10	0.105 (3)	0.060 (2)	0.082 (3)	−0.013 (2)	−0.005 (2)	−0.029 (2)
C11	0.0481 (19)	0.061 (2)	0.0616 (19)	−0.0093 (15)	−0.0014 (15)	−0.0259 (16)
C12	0.0605 (19)	0.0479 (17)	0.0505 (18)	−0.0071 (14)	−0.0080 (14)	−0.0016 (14)
C13	0.0554 (18)	0.0500 (17)	0.0438 (16)	−0.0113 (14)	0.0004 (13)	−0.0060 (13)
C14	0.064 (2)	0.0572 (19)	0.0470 (17)	−0.0053 (15)	−0.0085 (15)	−0.0100 (14)
C15	0.075 (2)	0.0523 (19)	0.0557 (19)	−0.0021 (16)	−0.0047 (16)	−0.0106 (15)
C16	0.064 (2)	0.066 (2)	0.0539 (19)	−0.0175 (17)	0.0086 (16)	−0.0187 (16)
C17	0.068 (2)	0.083 (3)	0.0450 (17)	−0.0144 (18)	−0.0014 (15)	−0.0196 (17)
C18	0.067 (2)	0.064 (2)	0.0445 (17)	−0.0026 (16)	−0.0013 (15)	−0.0045 (15)
C19	0.105 (3)	0.088 (3)	0.078 (3)	−0.020 (2)	0.006 (2)	−0.040 (2)
Br1	0.1216 (4)	0.0559 (3)	0.0471 (2)	−0.0034 (2)	−0.01539 (19)	−0.00359 (15)
O1	0.167 (3)	0.0439 (14)	0.0661 (16)	−0.0054 (15)	−0.0002 (17)	−0.0025 (12)
O2	0.0770 (15)	0.0477 (12)	0.0569 (13)	−0.0091 (10)	−0.0040 (11)	−0.0118 (10)
O3	0.0432 (11)	0.0475 (11)	0.0476 (11)	−0.0118 (8)	−0.0073 (8)	−0.0031 (9)
O4	0.0420 (13)	0.098 (2)	0.101 (2)	−0.0105 (12)	−0.0076 (13)	−0.0351 (16)

### Geometric parameters (Å, º)

**Table:**

C1—C2	1.388 (4)	C10—H10A	0.9600
C1—C6	1.399 (4)	C10—H10B	0.9600
C1—C11	1.461 (4)	C10—H10C	0.9600
C2—C3	1.365 (4)	C11—O4	1.203 (4)
C2—H2	0.9300	C11—H11	0.9300
C3—C4	1.376 (4)	C12—C13	1.459 (4)
C3—Br1	1.898 (3)	C12—H12	0.9300
C4—C5	1.381 (4)	C13—C14	1.387 (4)
C4—H4	0.9300	C13—C18	1.396 (4)
C5—C6	1.383 (4)	C14—C15	1.366 (5)
C5—H5	0.9300	C14—H14	0.9300
C6—O3	1.357 (3)	C15—C16	1.380 (5)
C7—O3	1.432 (3)	C15—H15	0.9300
C7—C8	1.496 (4)	C16—C17	1.369 (5)
C7—H7A	0.9700	C16—C19	1.501 (5)
C7—H7B	0.9700	C17—C18	1.360 (5)
C8—C12	1.328 (4)	C17—H17	0.9300
C8—C9	1.477 (4)	C18—H18	0.9300
C9—O1	1.196 (4)	C19—H19A	0.9600
C9—O2	1.330 (4)	C19—H19B	0.9600
C10—O2	1.442 (4)	C19—H19C	0.9600
			
C2—C1—C6	119.4 (3)	H10A—C10—H10C	109.5
C2—C1—C11	120.0 (3)	H10B—C10—H10C	109.5
C6—C1—C11	120.6 (3)	O4—C11—C1	124.4 (3)
C3—C2—C1	119.7 (3)	O4—C11—H11	117.8
C3—C2—H2	120.1	C1—C11—H11	117.8
C1—C2—H2	120.1	C8—C12—C13	130.8 (3)
C2—C3—C4	121.1 (3)	C8—C12—H12	114.6
C2—C3—Br1	120.1 (2)	C13—C12—H12	114.6
C4—C3—Br1	118.9 (2)	C14—C13—C18	117.1 (3)
C3—C4—C5	120.2 (3)	C14—C13—C12	124.8 (3)
C3—C4—H4	119.9	C18—C13—C12	118.1 (3)
C5—C4—H4	119.9	C15—C14—C13	120.8 (3)
C4—C5—C6	119.5 (3)	C15—C14—H14	119.6
C4—C5—H5	120.3	C13—C14—H14	119.6
C6—C5—H5	120.3	C14—C15—C16	121.7 (3)
O3—C6—C5	123.9 (2)	C14—C15—H15	119.1
O3—C6—C1	116.0 (2)	C16—C15—H15	119.1
C5—C6—C1	120.1 (3)	C17—C16—C15	117.6 (3)
O3—C7—C8	109.0 (2)	C17—C16—C19	120.8 (3)
O3—C7—H7A	109.9	C15—C16—C19	121.7 (3)
C8—C7—H7A	109.9	C18—C17—C16	121.6 (3)
O3—C7—H7B	109.9	C18—C17—H17	119.2
C8—C7—H7B	109.9	C16—C17—H17	119.2
H7A—C7—H7B	108.3	C17—C18—C13	121.2 (3)
C12—C8—C9	116.4 (3)	C17—C18—H18	119.4
C12—C8—C7	123.8 (3)	C13—C18—H18	119.4
C9—C8—C7	119.6 (3)	C16—C19—H19A	109.5
O1—C9—O2	121.9 (3)	C16—C19—H19B	109.5
O1—C9—C8	124.7 (3)	H19A—C19—H19B	109.5
O2—C9—C8	113.4 (3)	C16—C19—H19C	109.5
O2—C10—H10A	109.5	H19A—C19—H19C	109.5
O2—C10—H10B	109.5	H19B—C19—H19C	109.5
H10A—C10—H10B	109.5	C9—O2—C10	115.7 (3)
O2—C10—H10C	109.5	C6—O3—C7	117.3 (2)
			
C6—C1—C2—C3	−0.2 (4)	C6—C1—C11—O4	174.2 (3)
C11—C1—C2—C3	178.5 (3)	C9—C8—C12—C13	−180.0 (3)
C1—C2—C3—C4	−0.3 (4)	C7—C8—C12—C13	5.0 (5)
C1—C2—C3—Br1	179.79 (19)	C8—C12—C13—C14	30.2 (5)
C2—C3—C4—C5	0.2 (4)	C8—C12—C13—C18	−150.1 (3)
Br1—C3—C4—C5	−179.8 (2)	C18—C13—C14—C15	1.0 (5)
C3—C4—C5—C6	0.3 (4)	C12—C13—C14—C15	−179.3 (3)
C4—C5—C6—O3	178.7 (3)	C13—C14—C15—C16	−0.4 (5)
C4—C5—C6—C1	−0.7 (4)	C14—C15—C16—C17	0.1 (5)
C2—C1—C6—O3	−178.8 (2)	C14—C15—C16—C19	179.5 (3)
C11—C1—C6—O3	2.5 (4)	C15—C16—C17—C18	−0.5 (5)
C2—C1—C6—C5	0.7 (4)	C19—C16—C17—C18	−179.8 (3)
C11—C1—C6—C5	−178.0 (3)	C16—C17—C18—C13	1.1 (5)
O3—C7—C8—C12	−93.5 (3)	C14—C13—C18—C17	−1.4 (5)
O3—C7—C8—C9	91.6 (3)	C12—C13—C18—C17	178.9 (3)
C12—C8—C9—O1	0.9 (5)	O1—C9—O2—C10	−2.4 (5)
C7—C8—C9—O1	176.2 (3)	C8—C9—O2—C10	177.4 (3)
C12—C8—C9—O2	−178.9 (3)	C5—C6—O3—C7	3.3 (4)
C7—C8—C9—O2	−3.6 (4)	C1—C6—O3—C7	−177.2 (2)
C2—C1—C11—O4	−4.5 (4)	C8—C7—O3—C6	178.2 (2)

### Hydrogen-bond geometry (Å, º)

**Table:**

*D*—H···*A*	*D*—H	H···*A*	*D*···*A*	*D*—H···*A*
C14—H14···O3	0.93	2.55	3.341 (3)	143
C4—H4···O2^i^	0.93	2.57	3.474 (4)	164
C18—H18···O1^ii^	0.93	2.49	3.388 (4)	161
C5—H5···O4^iii^	0.93	2.39	3.276 (4)	159

Symmetry codes: (i) −*x*+1, −*y*, −*z*; (ii) −*x*+1, −*y*−1, −*z*+1; (iii) *x*+1, *y*, *z*.
